# OP53 An Actionable And Legible Toolbox For The Appraisal Of Healthcare Innovations Developed Through Nationwide Stakeholder Collaboration

**DOI:** 10.1017/S0266462324001156

**Published:** 2025-01-07

**Authors:** Geneviève Plamondon, Isabelle Ganache, Mélanie Martin, Pascale Lehoux

## Abstract

**Introduction:**

In Québec, Canada, decisions about implementing innovations are taken both centrally for province-wide access and locally by healthcare institutions. There is no systematic evaluation process and various stakeholders are involved, notably within a new nationwide governance structure. There was a wish to increase consistency and clarity with the principles and methods used by various bodies across the innovation lifecycle.

**Methods:**

The starting point was the Institut national d’excellence en santé et services sociaux (INESSS) multidimensional framework, which focuses on the population-level, clinical, economic, organizational, and sociocultural value of drugs, technologies, and interventions. The framework, already under evolution drawing on Responsible Innovation in Health (RIH), evolved through collaborative work between INESSS’ methodological and scientific teams, but also and foremost with diverse groups and institutions within the provincial innovation ecosystem (e.g., university-based incubators, regional hospitals). The first steps were to capture current concepts and practices from different stakeholders, as well as their operational needs in terms of assessment tools.

**Results:**

This multistakeholder taskforce resulted in the development of an operational toolbox meant to guide the value appraisal of innovations through a lifecycle approach. First aimed at stakeholders involved locally in healthcare institutions, the work conducted was equally beneficial to INESSS by enabling its evaluation teams to contribute to the operational tools needed to enhance clarity and legibility of the agency’s processes and methods. The level of collaboration with stakeholders across the province was also unique and has strengthened the understandability and actionability of the toolbox developed. Some challenges were faced, and related actions will be discussed.

**Conclusions:**

Both the taskforce process and its output contributed to improving consistency in the assessment of innovations across the province. They made more explicit what may sometimes be perceived as the HTA “black box.” The INESSS value appraisal framework also evolved considering key elements of responsibility from RIH and through this collaboration with stakeholders, and its applicability in different contexts was reinforced.

